# Depletion of CD52‐positive cells inhibits the development of central nervous system autoimmune disease, but deletes an immune‐tolerance promoting CD8 T‐cell population. Implications for secondary autoimmunity of alemtuzumab in multiple sclerosis

**DOI:** 10.1111/imm.12696

**Published:** 2017-01-03

**Authors:** Stephanie von Kutzleben, Gareth Pryce, Gavin Giovannoni, David Baker

**Affiliations:** ^1^ Neuroimmunology UnitBlizard InstituteBarts and the London School of Medicine & DentistryQueen Mary University of LondonLondonUK

**Keywords:** autoimmunity, experimental autoimmune encephalomyelitis/multiple sclerosis, neuroimmunology, tolerance/suppression/anergy

## Abstract

The objective was to determine whether CD52 lymphocyte depletion can act to promote immunological tolerance induction by way of intravenous antigen administration such that it could be used to either improve efficiency of multiple sclerosis (MS) inhibition or inhibit secondary autoimmunities that may occur following alemtuzumab use in MS. Relapsing experimental autoimmune encephalomyelitis was induced in ABH mice and immune cell depletion was therapeutically applied using mouse CD52 or CD4 (in conjunction with CD8 or CD20) depleting monoclonal antibodies. Immunological unresponsiveness was then subsequently induced using intravenous central nervous system antigens and responses were assessed clinically. A dose–response of CD4 monoclonal antibody depletion indicated that the 60–70% functional CD4 T‐cell depletion achieved in perceived failed trials in MS was perhaps too low to even stop disease in animals. However, more marked (~75–90%) physical depletion of CD4 T cells by CD4 and CD52 depleting antibodies inhibited relapsing disease. Surprisingly, in contrast to CD4 depletion, CD52 depletion blocked robust immunological unresponsiveness through a mechanism involving CD8 T cells. Although efficacy was related to the level of CD4 T‐cell depletion, the observations that CD52 depletion of CD19 B cells was less marked in lymphoid organs than in the blood provides a rationale for the rapid B‐cell hyper‐repopulation that occurs following alemtuzumab administration in MS. That B cells repopulate in the relative absence of T‐cell regulatory mechanisms that promote immune tolerance may account for the secondary B‐cell autoimmunities, which occur following alemtuzumab treatment of MS.

**Abbreviations**
EAE: experimental autoimmune encephalomyelitis
MS: multiple sclerosis
SCH: spinal cord homogenate

## Introduction

Multiple sclerosis (MS) is the major demyelinating disease of the central nervous system.^1^ Although the aetiology is obscure, genetic susceptibility, pathology and response to therapy indicate that the disease is immune‐mediated.^1^ Although MS appears to be uniquely human, clinical and pathological similarities between MS and experimental autoimmune encephalomyelitis (EAE) have resulted in MS being viewed as a T‐cell‐mediated autoimmune disease targeting oligodendroctyes.^1^, ^2^ Although the innate immune system and B cells can contribute to the disease process in EAE, it is clear that T‐cell activity is central to pathogenesis. This is indicated by the ability to adoptively transfer disease and via T‐cell inhibition.^2^, ^3^, ^4^ In many cases disease is mediated by CD4 T cells,^3^ although pathogenic CD8 T‐cell models have been developed to mirror the CD8 predominance in some MS lesions.^5^ However, supportive data for a CD4, T helper type 17‐mediated pathogenesis in MS is largely circumstantial and not supported by the perceived failure of CD4‐depleting monoclonal antibodies (mAb).^6^, ^7^, ^8^

In animals, the disease course is predictable allowing optimized treatment to achieve maximal inhibition and CD4 depletion can control most T‐ and B‐cell (T‐dependent) immune responses.^3^, ^4^, ^9^ However, at the time of the initial CD4 mAb trials, AIDS was becoming prevalent as a result of HIV. Therefore, long‐term depletion below 250 CD4 cells/mm^3^ (about 70% depletion) was felt to be contra‐indicated and trials aimed to maintain CD4 T‐cell numbers above this limit.^6^, ^10^ This is substantially less than the > 85% depletion used to inhibit EAE.^3^, ^4^ However, alemtuzumab, which is a CD52 lymphocyte‐depleting mAb, produces a long‐term and marked (> 90%) depletion of CD4 T cells and effectively inhibits relapsing MS.^11^, ^12^ However, secondary B‐cell autoimmune diseases often occur as a delayed side‐effect of alemtuzumab treatment in people with MS.^11^, ^12^, ^13^ In addition, generalized immunosuppression may result in infections and other adverse effects^12^, ^13^, ^14^ that may limit the wide adoption of the treatments.

Antigen‐specific immunotherapy has the advantage of controlling pathogenic T cells while leaving the rest of the immune system to fight infection and cancers. Although there are many ways to induce antigen‐specific tolerance, a consistently robust method has been achieved by intravenous antigen delivery following transient T‐cell deletion.^4^, ^15^ This combination, and not the individual treatments, eliminates relapsing EAE in animals with established disease.^4^ Similarly, depletion of CD4 T cells^6^, ^7^ and depletion of intravenous oligodendrocyte‐directed antigens^16^, ^17^ have been tried and so far failed to eliminate relapses in MS, despite some efficacy. However, these data indicate that such combinations could be safe and feasible in MS. No CD4 mAb is currently licenced for MS so it was hypothesized that alemtuzumab could be used as a T‐cell‐depleting agent for tolerance induction. This could perhaps be used in combination with oliogodendrocyte‐associated autoantigens to control MS or other autoantigens, such as thyroid antigens to treat the alemtuzumab‐induced autoimmune diseases that are highly prevalent in MS.^11^, ^12^, ^13^, ^14^ This hypothesis was investigated using a mouse CD52‐specific mAb in relapsing EAE.

## Materials and methods

#### Animals

Male or female Biozzi ABH mice were from stock bred at Queen Mary University of London, these were housed and fed with RM1(E) chow and water *ad libitum* as described previously.^18^ They were used according to the United Kingdom, Animals (Scientific procedures) Act 1986, incorporating review by the local Animal Welfare and Ethical Review Body and the United Kingdom Home Office.

#### Antibodies

Purified and fluorescent mouse CD4 (mCD4) ‐specific mAb were used: rat IgG2b clone YTS191.1 mAb (Bio X cell, West Lebanon NH; AbD Serotec Kidlington, UK); rat IgG2b RM4‐5 (AbD Serotec); rat IgG2b clone YTA3.1 (Dr S. Cobbold, University of Oxford), rat IgG2b GK1.5 (AbD Serotec); rat IgG2c KT174 (AbD Serotec and Dr K. Tomonari, Fukui Medical School, Japan) or rat IgG2a KT6 (Dr K. Tomonari) were obtained.

#### In vivo *mAb treatment*

Mice were injected intraperitoneally or subcutaneously with various amounts of, typically 250 μg, mCD4‐depleting rat IgG2b (YTS191.1) mAb.^3^, ^4^ This was purchased from Bio X cell. Genzyme Corporation (Framingham MA) supplied the 250 μg mouse CD52 (mCD52) ‐depleting mouse IgG2a mAb.^19^ This was injected subcutaneously over 5 consecutive days.^20^ Mouse 18B12 CD20‐depleting mouse IgG2a mAb^21^ was a kind gift of Biogen Idec Inc. (Cambridge, MA). Irrelevant rat IgG2b and mouse IgG2a mAbs do not influence the course of EAE in ABH mice.^3^, ^18^

#### Flow cytometry

Erythrocyte‐free splenocytes generated using erythrocyte lysis buffer (eBiosciences Ltd, Hatfield, UK) were counted and then 1 × 10^6^splenocytes were incubated with 1–5 μg/ml fluorescent mouse‐specific antibodies. CD4 and CD8 T cells were identified using CD4‐phycoerythrin and CD8a‐phycoerythrin‐Cy7 conjugates, respectively. Allophycocyanin‐conjugated CD45RA (B220. RA3‐6B2) was used to identify B cells, CD335 Peridinin chlorophyll protein‐Cy5 identified natural killer cells and neutrophils were stained using GR‐1 allophycocyanin‐Cy7. These were purchased from Becton Dickinson (Oxford, UK). Monocytes/macrophages were characterized by the binding of F4/80 FITC (eBiosciences). Regulatory T cells were detected using: CD4 FITC, CD25 allophycocyanin‐Cy7 (both Becton Dickinson) and intracellular Forkhead box P3‐phycoerythrin (FJK‐16s; eBiosciences). Staining of regulatory T cells was performed according to the manufacturer's protocol. Briefly, following the extracellular staining, cells were incubated with a fixation/permeabilization working solution for at least 30 min at 4° in the dark. The cells were then centrifuged at 300 ***g*** for 3 min, washed with permeabilization buffer (prepared from a 10× stock solution) and centrifuged once more. Intracellular antibodies, including isotype controls, were added at appropriate dilutions in permeabilization buffer with 5% mouse serum and incubated for 30 min at 4° in the dark. The cells were then washed and resuspended in FACS buffer before flow cytometric analysis. The lymphocyte population was gated on forward, side‐scatter characteristics. In some instances, splenocytes were pre‐incubated with saturating 20 μg/ml amounts of unconjugated CD4‐specific mAb, for 30–60 min before incubation with conjugated CD4‐specific mAb.

#### Induction of experimental autoimmune encephalomyelitis

Six‐ to eight‐week‐old adult ABH mice were subcutaneously injected with 1 mg mouse spinal cord homogenate (SCH) emulsified in Freund's complete adjuvant containing 60 μg *Mycobacterium tuberculosis* H37Ra and *Mycobacterium butyricum* (8 : 1) in the flank on days 0 and 7 as described previously.^18^ Clinical disease was scored: Normal = 0; Fully flaccid tail = 1; Impaired righting reflex = 2; Hindlimb paresis = 3; Complete hindlimb paralysis = 4 and Moribund/death = 5.^18^ Details of randomization, blinding and sample size calculations and other experimental details relevant to the ARRIVE guidelines have been reported previously.^18^ Use of SCH as immunogen precludes *ex vivo* analysis as SCH‐sensitized animals fail to give robust T‐cell responses to the pathodominant myelin epitopes; however, the mechanisms of unresponsiveness induced by intravenous antigen delivery have been described previously.^4^, ^15^ The data are typically plotted as a Kaplan–Meirer curve to allow animals to be removed from the study, rather than remain with disability and hence offers advantage in the Refinement, Reduction and Replacement (3Rs) of animals in research.

#### Induction of unresponsiveness

Erythrocyte‐free splenocytes were prepared from ABH mice and SCH was chemically coupled to splenocytes using 1‐ethyl‐3‐(3‐dimethylaminopropyl) carbodiimide for 1 hr as described previously^18^ and 2·5 × 10^7^ SCH–antigen coupled spleen cells (SCH‐SC) in 0·1–0·2 ml of PBS were injected intravenously into the tail vein of each mouse.^18^ This was administered 1–3 weeks after CD4 T‐cell depletion. To assess the development of unresponsiveness, animals were rechallenged with a further set of injections of SCH in Freund's incomplete adjuvant typically 2 weeks after tolerance induction.^4^

#### Statistical analysis

Results represent the mean maximum ± SEM clinical score or day of onset ± SD, and were analysed using non‐parametric statistics using sigmaplot V11.^18^

## Results

### Repopulation kinetics and immune inhibitory function following CD4 T‐cell depletion

**Figure 1:**
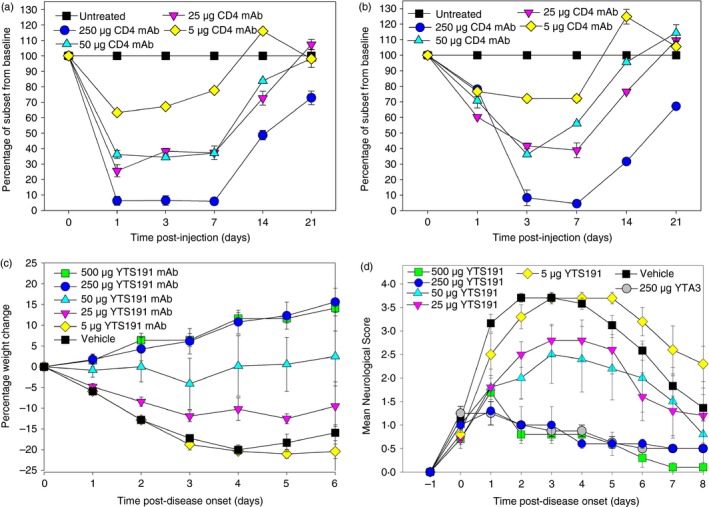
Dose response of physical depletion and receptor blockade the ability to control the development of experimental autoimmune encephalomyelitis (EAE) by CD4 T‐cell (YTS191.1) depleting monoclonal antibody (mAb). Animals were injected with 250 μg YTS191.1 CD4‐depleting mAb on day 0. Splenocytes removed were stained with: (a) FITC‐conjugated YTS191.1 or (b) phycoerythrin‐conjugated RM4.4 CD4‐specific mAb and analysed by flow cytometry. The results represent the mean ± SEM per cent of cells *n* = 3/group. (c, d) Animals were injected with 1 mg spinal cord homogenate in Freund's complete adjuvant on day 0 and 7. Following the onset of neurological disease animals were randomly assigned to treatment with various doses of YTS191.1 CD4‐depleting mAb or YTA3.1 and these were (c) weighted and the (d) neurological score was assessed. The results represent the mean ± SEM (*n* = 8–10/group). [Colour figure can be viewed at wileyonlinelibrary.com]

Previously it has been reported that physical depletion of CD4 T cells can inhibit disease and 250 μg of YTS191.1 antibody silenced CD4 T‐cell activity for approximately 3 weeks following detection of the same CD4 epitope used for depletion.^3^ Cross‐blocking experiments indicated that there are a number of distinct CD4 epitopes that can be used to monitor T‐cell depletion with YTS191.1 rat IgG2b monoclonal antibody (see Supplementary material, Fig. S1, Table S1). Following administration of 250 μg rat IgG2b CD4‐depleting (YTS191.1) mAb there was rapid receptor blockade of ~90% cells within 24 h (Fig. 1a). However, by 14 days lymphocytes had recovered to about 50–70% of their original values at a time when there was limited rat immunoglobulin detected. This is achieved by rapid expansion of the memory T‐cell pool.^4^ The level of receptor blocked with 25 μg and 50 μg YTS191.1 CD4‐specific mAb was largely comparable and created a functional depletion of 60–70% (Fig. 1a), but 5 μg CD4‐depleting mAb had minimal impact and reduced the level of CD4^+^ T cells by ~30% for 1 week (Fig. 1a). The pattern of depletion as identified using antibodies targeting the RM4.4 antigen was similar to the YTS191.1 epitope, except that it was clear that physical depletion was taking longer than the receptor blockade (Fig. 1b). At 1 day after administration of 250 μg YTS191.1 intraperitoneally 15·4 ± 0·9% of the splenocytes exhibited surface rat immunoglobulin but this dropped to 1·84 ± 0·1% at 3 weeks after administration, when CD4 had already begun to recover. Physical depletion of CD4 T cells, as indicated by the number of cells expressing the RM4‐4 epitope was ~90%, but by 3 weeks the number of cells had recovered to be about 30% of the splenic population. Both 25 μg and 50 μg YTS191.1 CD4‐specific mAb induced a similar level of depletion that peaked with about a 60% depletion within 3 days and with a 20–30% depletion at 2 weeks following antibody administration. There was essentially no depletion 27 days after antibody administration. Five micrograms of CD4‐depleting mAb had minimal impact and reduced the level of CD4^+^ T cells by a maximum of ~30% (Fig. 1b). This level of depletion failed to influence the development of EAE when animals were randomized to treatment following the onset of EAE when they were exhibiting a paralysed tail and weight loss (Fig. 1c) and the development of neurological disease (Fig. 1d). Inhibition of EAE‐associated weight loss was dose‐dependent and it was clear that both 500 μg and 250 μg YTS191.1 mAb controlled weight loss and animals began to recover within 24 h following injection. Although weight loss was stabilized following injection of 50 μg YTS191.1 mAb, neurological disease continued to deteriorate. Likewise although there was a small amelioration of disease activity, animals treated with 25 μg YTS191.1 mAb continued to develop disease (Fig. 1d). Therefore, in this highly optimized system, depletion of CD4 numbers by about 50–60% was insufficient to effectively control disease in EAE and about 30% CD4 T‐cell depletion exhibited no control of EAE. Similar inhibition of neurological EAE was achieved following injection of 250 μg YTA3.1 (Fig. 1d).

### CD4 T‐cell depletion can inhibit relapsing EAE

**Figure 2:**
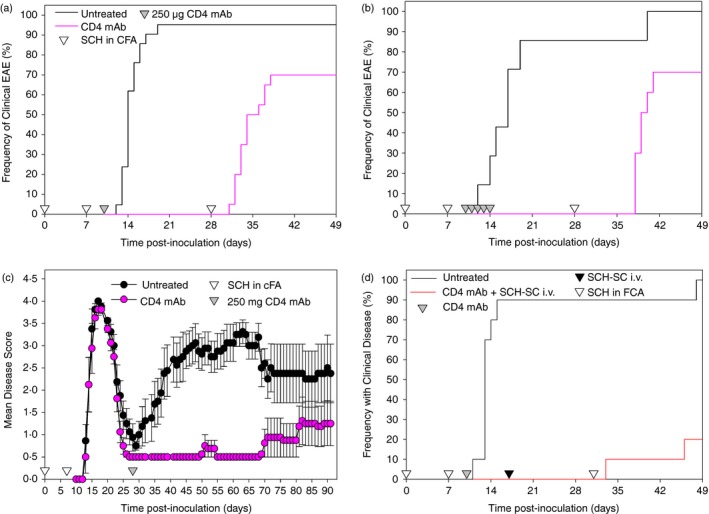
CD4 T‐cell depletion inhibits the development of relapsing experimental autoimmune encephalomyelitis (EAE). Biozzi ABH mice were injected with mouse spinal cord homogenate (SCH) in Freund's complete adjuvant on days 0 and 7 (open inverse triangles). (a) They were left untreated or received: (a) a single 250 μg YTS191.1 intraperitoneal injection of YTS191.1 CD4‐depleting mAb on day 10 post‐inoculation (p.i.) (*n* = 20–21) or (b) over 5 consecutive days from day 10 to day 14 p.i. All mice were rechallenged with SCH in Freund's complete adjuvant on day 28 (*n* = 10). Results indicate the day of onset of clinical EAE for each animal (c). Animals received a single 250 μg YTS191.1 mAb intraperitoneally on day 28 p.i. The results represent the mean ± SEM daily neurological score (*n* = 8–10 mice per group) (d) Animals were injected 250 μg YTS191.1 intraperitoneally on day 10 and 2 × 10^7^ SCH‐SC intravenously on day 17. Animals were rechallenged with SCH in Freund's incomplete adjuvant on day 31. The results indicate the day of onset of clinical EAE for each animal. [Colour figure can be viewed at wileyonlinelibrary.com]

Two hundred and fifty micrograms YTS191.1 mAb could inhibit the development of EAE in Biozzi ABH mice when administered as a single injection on day 10 post‐inoculation (p.i.) (Fig. 2a). Control mice developed EAE with high incidence and mice that developed disease exhibited a mean maximum neurological score of 3·7 ± 0·2 compared with CD4‐T‐cell‐depleted mice that exhibited no disease, Score 0·0 ± 0·0 (*P* < 0·001) up to day 24 p.i. Freedom from disease was maintained until animals were rechallenged with a further injection of antigen in Freund's incomplete adjuvant on day 28 p.i. where the majority of animals (*n* = 14/20) developed disease (Fig. 2a). This procedure can reactivate previously primed cells in an antigen/peptide‐specific manner and breaks unresponsiveness induced by either CD4 T‐cell depletion or intravenous antigen alone but not the combination of CD4 depletion and intravenous antigen.^4^ In this experiment, control animals relapsed and exhibited mean maximal score of 3·5 ± 0·2 in the animals developing disease (*n* = 20/21) and was not significantly different from the animals developing disease after rechallenge where the mean maximal score was 3·9 ± 0·1. Increasing the number of doses did not improve this whereas five daily subcutaneous CD4‐mAb administrations of YTS191.1 could again completely inhibit the development of EAE. However, it again failed to limit the capacity to re‐induce EAE, indicating that robust immune unresponsiveness, which can resist antigen rechallenge, via multiple mechanisms,^4^, ^18^, ^21^ was not induced (Fig. 2b). Similar to inhibition of the initial acute EAE attack, relapsing disease was inhibited when 250 μg CD4‐depleting mAb was injected during the post‐acute remission phase with 0/8 animals relapsing up to day 60 p.i. compared with 8/8 in untreated controls (Fig. 2c). However, with time, disease began to develop and by day 90 p.i. it was found that 3/8 CD4‐treated animals had relapsed (mean day of onset 66·7 ± 15·6 compared with controls day 36·7 ± 5·3 (*P* < 0·01) (Fig. 2c). However, when CD4 T‐cell depletion was combined with intravenous antigen in the form of 2·5 × 10^7^ SCH‐coupled splenocytes (SCH‐SCH), robust unresponsiveness was induced that could largely (*n* = 2/10 *P* < 0·001) resist antigen rechallenge (Fig. 2d).

### Repopulation kinetics and immune inhibitory function following CD52 cell depletion

**Figure 3:**
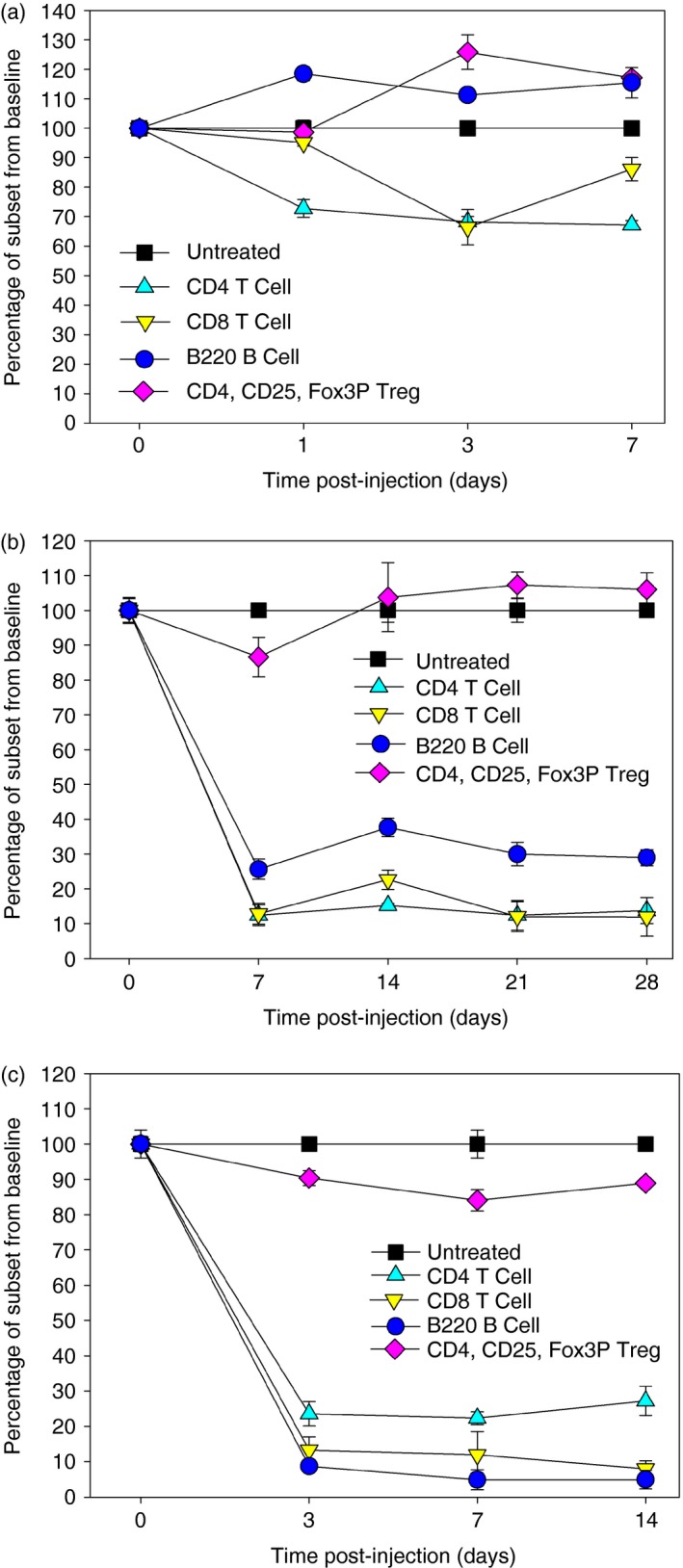
Depletion of leucocyte subsets by a mouse CD52‐specific monoclonal antibody (mAb) in ABH mice. Animals were injected with either: (a) a single injection (*n* = 3) or (b, c) five consecutive daily injections (*n* = 3/group) of 250 μg mouse CD52‐specific mAb. (a, b) Splenocytes or (c) peripheral blood leucocytes were prepared and stained with directly conjugated fluorescent CD4 (clone RM4.4); CD8, CD45RA/B220 or a combination of CD4, CD25 and Fox3P‐specific antibodies. Cells were analysed by flow cytometry. The results represent the mean ± SEM per cent of cells compared with the absolute cell number at baseline. [Colour figure can be viewed at wileyonlinelibrary.com]

The pattern of splenic lymphocyte repopulation following CD52 depletion was assessed in ABH mice by flow cytometry. Surprisingly, following a single injection of 250 μg (10 mg/kg) mouse CD52‐specific mAb on day 10 there was only about a 30% CD4 T‐cell depletion (Fig. 3a); this contrasts with the marked depletion of this splenic population by CD4‐specific mAb (Fig. 1a,b). As alemtuzumab is administered as a set of five daily infusions in humans,^12^ five daily subcutaneous injections of mouse CD52‐mouse IgG2a mAb^19^ were administered according to instructions from the manufacturers. It was found that the relative percentages of both CD4 and CD8 T cells were now depleted by approximately 80–90% within 7 days of antibody delivery and this level of depletion persisted during an observation period of 28 days (Fig. 3b). CD4, CD25, FoxP3 positive T regulatory cells were relatively unaffected by CD52‐mAb. There, was also marked depletion of CD45RA/B220 B cells within the spleen (70 ± 0·6%; Fig. 3b). However, there was substantially (*P* < 0·05) more B‐cell depletion (95·9 ± 0·1%. *n* = 3) within the peripheral blood, than within the spleen, within 7 days of initiating antibody treatment (Fig. 3c). This compared to 88·0 ± 0·1% (spleen) and 88·1 ± 0·2 (blood) depletion of CD8 T cells.

### Inhibition of relapsing EAE with CD52‐specific mAb

**Figure 4:**
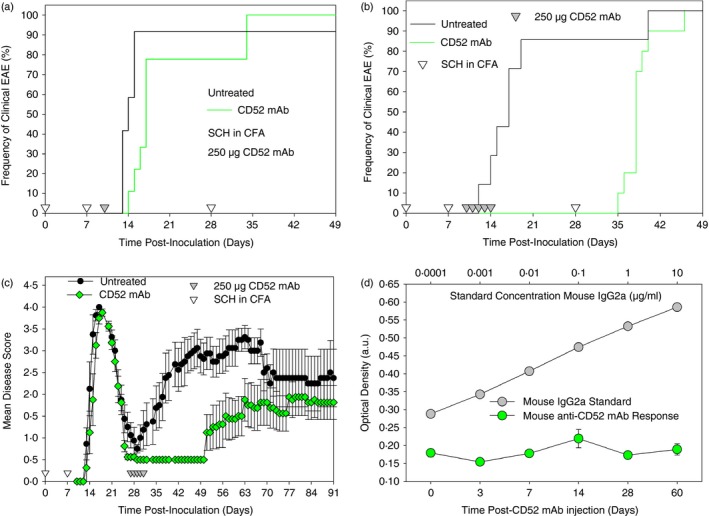
CD4 T‐cell depletion inhibits the development of relapsing experimental autoimmune encephalomyelitis (EAE). (a–c) Biozzi ABH mice were injected with mouse spinal cord homogenate (SCH) in Freund's complete adjuvant on days 0 and 7 (open inverse triangles). (a) They were left untreated or received: (a) a single subcutaneous 250 μg CD52 monoclonal antibody (mAb) injection on day 10 post‐inoculation (p.i.) (*n* = 10/group) or (a, b) 250 μg CD52 mAb each day for 5 consecutive days from day 10 to day 14 p.i. All mice were rechallenged with SCH in complete Freund's adjuvant on day 28 (*n* = 10/group). Results indicate the day of onset of clinical EAE for each animal in a Kaplan–Meier plot. Untreated control reuslts are displayed in black (c). Animals received five consecutive subcutaneous injections of 250 μg CD52 mAb on day 28–32 p.i. The results represent the mean ± SEM daily neurological score (*n* = 8/group) (d). Serum was periodically collected from ABH mice treated with the 5‐day course of mouse CD52‐specific mAb (open circles). The presence of anti‐CD52 antibodies was analysed by ELISA using plates coated with 4 μg/ml mouse CD52‐specific mAb and incubated with 1 : 100 dilutions of serum for 1 h, which was detected using horseradish peroxidase (HRP) ‐conjugated rabbit anti‐mouse IgG1. Serial dilutions of a rabbit anti‐mouse IgG2a antibody were exposed to plates coated with mouse CD52‐specific mAb. Binding was detected using HRP‐conjugated goat anti‐rabbit IgG. All plates were developed using ABTS and absorbance was measured at 410 nm. Results represent the mean optical density ± SEM of each time point (*n* = 3 mice per time point assessed). [Colour figure can be viewed at wileyonlinelibrary.com]

It was found that consistent with the low level (~30%) of CD4 T‐cell depletion induced by the CD52 mAb (Fig. 3a), a single injection of 250 μg CD52 mAb failed to prevent the development of EAE (Fig. 4a). In contrast, repeated subcutaneous administrations of 250 μg CD52 mAb, which depleted splenic CD4 T cells by ~90% (Fig. 4a–c), completely inhibited the development of disease *n* = 0/10 (*P* < 0·001; Group Score 0·0 ± 0·0 *P* < 0·001) compared with control mice that developed (*n* = 6/7) severe EAE (Group score 3·5 ± 0·6 and EAE Score 4·1 ± 0·3 and Day of onset 16·7 ± 2·5) up to day 24 p.i. (Fig. 4b). However, as with the depletion induced by CD4‐specific mAb, disease could be reactivated following a further antigen‐rechallenge with SCH in Freund's incomplete adjuvant administered within about 2 weeks of cessation of antibody treatment (Fig. 4a,b). This indicates that disease can be induced despite significant T‐cell depletion. Therefore, mCD52d does not appear to re‐induce immunological unresponsiveness to autoimmune central nervous system disease as shown here. This effect was compatible with the efficacy of a single injection of 250 μg mCD4d mAb, reported previously.^3^, ^4^ It was found that control animals relapsed (*n* = 5/5 as two animals were culled during the initial acute attack) and exhibited severe disease (Group score 4·0 ± 0·0) that developed 9·0 ± 2·8 days after antigen rechallenge. This was not significantly different for mCD52‐depleting mAb where all animals developed disease (*n* = 10/10) with a Group Score of 3·6 ± 0·1 that developed 11·6 ± 3·0 days after rechallenge (Fig. 4b). In addition to inhibition of the initial acute phase of EAE (Fig. 4b), likewise, following the establishment of EAE, daily subcutaneous administration of 250 μg mCD52d mAb from day 27–31 p.i. inhibited the incidence (*P* = 0·002) and development (*P* = 0·001) of relapse. As such, no animal (*n* = 0/8) had developed a relapse by day 50 p.i. compared with 7/8 controls (*P* = 0·002) (Fig. 4c). However, this inhibitory effect was transient and spontaneous relapse eventually developed (*n* = 5/8 CD52‐specific mAb versus controls *n* = 8/8, maximum relapse score 3·4 ± 0·4 versus 4·0 ± 0·0 in controls) about 3 weeks after the last antibody administration, which was later (Day of relapse onset 56·4 ± 5·4 days in mCD52‐treated animals versus 36·7 ± 5·3 days in controls) than observed in untreated controls representing a significant (*P* < 0·001) delay in the onset of spontaneous relapse when observed until day 80 p.i. Therefore, although the mCD52d mAb was immunosuppressive, it did not induce robust immunological unresponsiveness.

In MS binding and inhibitory responses to alemtuzumab can develop within a month of infusion.^22^, ^23^ Although the mCD52‐specific mAb is of mouse origin, as ABH mice do not express the *Igh1a* allotype associated with production of IgG2a isotype and largely produce antibodies of the IgG1 and IgA isotypes,^24^, ^25^ the generation of IgG allotypic/idiotypic antibodies was examined, but was not detected when examined up to 2 months after administration (Fig. 4d).

### Mouse CD52‐specific mAb inhibits the generation of immunological unresponsiveness

**Figure 5:**
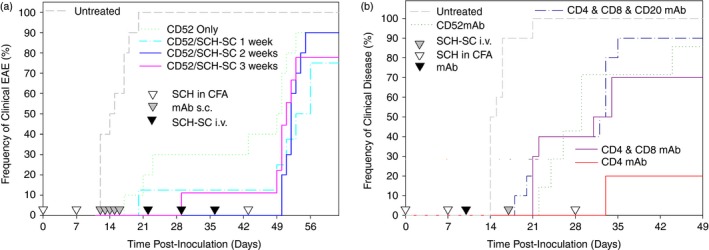
The combination of CD52‐mediated depletion and intravenous antigen does not induce myelin‐specific immune unresponsiveness in ABH mice. Experimental autoimmune encephalomyelitis (EAE) was induced in ABH mice by injection of spinal cord homogenate (SCH) in Freund's complete adjuvant on days 0 and 7. (a) On day 12 post‐inoculation (p.i.), mice were untreated or were injected subcutaneously with 250 μg mouse CD52‐specific monoclonal antibody (mAb) daily for 5 consecutive days. CD52 mAb animals were untreated or injected intravenously with 2·5 × 10^7^ antigen‐coupled splenocytes (SCH‐SC) at 1, 2 and 3 weeks after the last CD52 mAb injection. Disease was re‐induced in all animals on day 43 p.i. with SCH in Freund's incomplete adjuvant. Results represent the time of onset of disease for each animal from initial immunization. (*n* = 7–10 mice per group) (b). ABH mice were inoculated with SCH in CFA on days 0 and 7. At day 10 p.i., mice were injected with either 250 μg mouse CD52‐specific mAb days 10–14, or a single injection of 250 μg mouse YTS191.1 CD4‐specific mAb, or a combination of either 250 μg CD4 and 250 μg CD8‐specific (YTS169.4) mAb, or 250 μg CD4, 250 μg CD8 and 250 μg CD20‐specific (18B12) mAb intraperitoneally. On day 17 p.i., mice were injected intravenously with 2·5 × 10^7^ SCH‐coupled splenocytes (SCH‐SC). Disease was re‐induced at day 28. Untreated animals are shown in black. Results represent the time of onset of disease from initial immunization for each animal (*n* = 7–10 mice per group). [Colour figure can be viewed at wileyonlinelibrary.com]

To determine whether mCD52d mAb could facilitate intravenous antigen‐specific tolerance induction as shown with CD4‐depleting mAb (Fig. 2d),^4^ EAE was inhibited following daily subcutaneous administrations of 250 μg mCD52‐depleting from day 10 to day 14 p.i. (Fig. 5a). Intravenous antigen in the form of 2·5 × 10^7^ SCH coupled to splenocytes (SCH‐SC i.v.) was administered 1 week after the initiation of mAb treatment on day 17 and then animals were rechallenged with SCH 2 weeks after SCH‐SC i.v. to assess tolerance induction. The majority of animals (8/10) developed disease (Group score 2·9 ± 1·8 and EAE score 3·8 ± 0·1 in CD52‐specific mAb‐treated animals) compared with 10/10 animals developing EAE (Group score/EAE 3·6 ± 0·1) about a week after rechallenge (Day of onset 11·6 ± 3·0 in CD52‐specific mAb‐treated animals versus 10·8 ± 2·0). To counter the argument that insufficient cell numbers were present at the time of SCH‐SC i.v. administration to allow unresponsiveness to develop, tolerance induction was therefore assessed at 1 (day 22 p.i.), 2 (day 29 p.i.), and 3 (day 36 p.i.) weeks after the final dose of CD52‐specific mAb (Fig. 5a). Animals treated with CD52‐specific mAb typically displayed no clinical signs of disease until disease re‐induction was attempted at day 43 (Fig. 5a). Although such treatment delayed disease development (*P* < 0·001) (Table 1), by the termination of the experiment, the majority of mice had developed clinical EAE, regardless of treatment strategy; with 70–90% of animals treated with CD52‐specific mAb alone or in combination with intravenous myelin antigens developing EAE (Fig. 5a, Table 1). Furthermore, there was no significant difference in either the mean maximum disease score between groups or the animals that specifically developed within each group (Table 1). This suggests that this CD52‐specific mAb was not able to induce immune unresponsiveness, and in comparison to CD4‐specific mAb, it could perhaps be blocking tolerance induction by deleting a regulatory cell population.

**Table 1:** The combination of CD52‐mediated depletion and intravenous antigen does not induce immune unresponsiveness in ABH mice

Group	No.EAE/Total	Group max. score ± SEM	EAE max. score ± SEM	Mean day of onset ± SD
Untreated	10/10	3·9 ± 0·1	3·9 ± 0·1	16·3 ± 3·9
CD52 mAb	9/10	3·2 ± 0·4	3·6 ± 0·1	50·2 ± 1·5***
CD4mAb + 1 week SCH‐SC	5/7	2·5 ± 0·7	3·5 ± 0·3	54·0 ± 1·4***
CD4mAb + 2 weeks SCH‐SC	9/10	3·2 ± 0·4	3·5 ± 0·2	53·1 ± 1·7***
CD4mAb + 3 weeks SCH‐SC	6/8	2·8 ± 0·6	3·7 ± 0·2	48·7 ± 8·4***
Untreated	10/10	3·9 ± 0·1	3·9 ± 0·1	16·4 ± 2·2
CD4mAb + SCH‐SC	2/10	0·6 ± 0·4***	3·0 ± 0·5	34·0 ± 0·0***
CD4 and CD8 mAb + SCH‐SC	7/10	2·7 ± 0·6	3·9 ± 0·1	27·3 ± 6·4***
CD4,CD8 and CD20 + SCH‐SC	9/10	3·2 ± 0·4	3·5 ± 0·2	28·4 ± 6·9***

Experimental autoimmune encephalomyelitis (EAE) was induced in ABH mice by injection of spinal cord homogenate (SCH) in Freund's complete adjuvant on days 0 and 7. On day 12 post‐inoculation (p.i.), mice were untreated or were injected subcutaneously with 250 μg mouse CD52‐specific monoclonal antibody (mAb) daily for 5 consecutive days. CD52 mAb animals were untreated or injected intravenously with 2·5 × 10^7^ antigen‐coupled splenocytes (SCH‐SC) at 1, 2 and 3 weeks after the last CD52 mAb injection. Disease was re‐induced in all animals on day 43 p.i. with SCH in Freund's incomplete adjuvant. Results represent the number of animals that developed disease, the mean day of onset ± SD for each animal from initial immunization for the first attack, the mean ± SEM maximum neurological score of the first attack of all animals in each group (Group Score), the mean ± SEM maximum neurological score of the first attack of animals that developed EAE in each group (EAE Score) and the mean ± SD day of onset of disease (***P < 0·001 between treatment and control).

The induction of unresponsiveness was assessed by supplementing the CD4‐specific mAb‐induced depletion using CD8 (YTS169.4)^3^ and CD20 (18B12)^20^ depleting mAb. As shown previously, 250 μg CD8‐depleting antibody induces marked depletion^3^ and fails to prevent the development of EAE, such that it behaves as the untreated control in terms of incidence, onset and severity.^3^ Although CD4 mAb and intravenous antigen induced unresponsiveness that could largely resist an antigen rechallenge as shown previously,^4^ it was found that depletion of CD4‐ and CD8‐positive T cells or CD4, CD8 and CD20 cells, developed disease that was comparable to that seen with CD52‐mediated depletion (Fig. 5b). The majority (70–90%) of animals treated with CD8‐specific mAb developed paralytic disease, supporting the concept that a population of regulatory CD8 T cells were controlling unresponsiveness during EAE. This population could be inhibited as a consequence of CD52‐mediated depletion and so could perhaps contribute the occurrence of secondary autoimmune disease that occurs following use of alemtuzumab in MS.

## Discussion

This study further supports the view that central nervous system autoimmunity is mediated by the action of CD4 T cells. This molecule appears to contain at least four different domains that can be detected by distinct CD4 mAb clones.^26^, ^27^ These were used to demonstrate that CD4 mAb can both functionally, by blocking the receptor, and physically deplete T cells to inhibit disease. In young adult mice, CD4 T‐cell depletion lasted about 2–3 weeks before returning to about 30% of their original levels, which was not sufficient to control autoimmunity.^3^, ^4^ It was found that a functional depletion about 70% of baseline levels is needed to inhibit disease in an optimized animal system. In humans studies, CD4 depletion mAb also only induced a transient deletion and T‐cell numbers also rapidly repopulated within a month^10^ followed by a persistent lymphopenia for many months.^10^, ^25^, ^28^ Furthermore, as found in mice also,^4^ this treatment targeted the naive CD4 T‐cell subsets and there was particularly rapid repopulation of the CD40RO memory T‐cell subset,^28^, ^29^ which would contain the pathogenic T cells. It was found that depletion of T cells by about 50–60% failed to stop EAE from developing in this optimized system. This contrasts with the marked 70–95% depletion of CD4 T cells induced by alemtuzumab.^11^ This level of depletion by CD4‐specific mAb, including the lack of targeting of CD45RO^+^ T cells, suggests that the trials in MS were unlikely to have succeeded.^6^ This perceived failure questioned the role of CD4 T cells in MS and also supported the devaluing of the use of animal models of MS.^8^ However, such a failure could have been predicted if a few simple experiments had been performed before embarking on a clinical trial programme. This represents a further example of how human activity and trial design, rather than animal studies, probably contributed to the failure to translate.^2^ Interestingly, however, CD4‐specific mAb were not inert and there was a modest and significant reduction in relapse rate in MS.^6^ However, despite the suggestion that efficacy was related to T‐cell number;^6^ maintenance of depleted levels of T cells were not attempted further, until alemtuzumab was used.

Alemtuzumab targets CD52‐expressing cells and produces rapid, marked (< 200 cells/mm^3^) and sustained CD4 and CD8 T‐cell depletion lasting many months, whereas there is rapid repopulation of CD19^+^ B cells to levels above baseline.^11^ Importantly, this resulted in the inhibition of lesion formation and relapsing disease and alemtuzumab is currently one of the most effective licensed treatments of MS.^12^ The effectiveness of alemtuzumab is used to support the concept that T cells drive relapsing disease, which can be clearly shown in animal models. It is therefore of interest that it has been reported that disease activity can sometimes be associated with CD4^+^ T‐cell reconstitution,^30^, ^31^ such that counts below 390 CD4 T cells/mm^3^ (about 55–60% depletion) predicted stability.^30^ This is consistent with the hypothesis that a threshold of T‐cell depletion is required to effectively target the pathogenic T‐cell pool. The data obtained from CD4‐depleting mAb trials in MS may therefore provide only a weak argument against T cells being important in MS and indeed long‐term and marked (95–70%) depletion of CD4‐ and CD8‐positive T cells with alemtuzumab are associated with inhibition of the development of relapses in MS.^12^

In this study mCD52‐depleting mAb also depleted T cells and inhibited the development of relapsing EAE, which is associated with reduced immune infiltration and consequent reduced demyelination and neuroprotection.^2^, ^19^ The depletion profiles appeared relatively similar to that seen in hCD52 transgenic CD‐1 mice injected with alemtuzumab and SJL and C57BL/6 mice injected with the mCD52‐depleting mAb.^19^, ^32^, ^33^ However in contrast to proteolipid protein‐induced relapsing EAE in SJL mice,^19^ inhibition of EAE was more transient and disease often returned, as has been seen after cessation of treatment with immunosuppressive agents.^4^, ^34^ Furthermore, disease onset could be accelerated following reactivation of antigen‐specific primed cells with an antigen rechallenge, which can occur in both athymic and euthymic mice following CD4 T‐cell depletion.^4^ Therefore, there is some variability in responsiveness of individual mouse models related to depletion status and it is clear that there is some variability in the responsiveness of people to alemtuzumab.^13^

The use of animals also permits the investigation of tissues typically inaccessible in humans, and differences were found between the peripheral blood and secondary lymphoid organs. Consistent with other studies,^32^, ^33^ there was less marked depletion of B cells within the lymphoid tissues compared with the peripheral blood compared with T cells.^32^, ^33^ This may relate to the mechanism of depletion of alemtuzumab that centres on antibody‐dependent cellular cytotoxicity rather than complement fixation.^32^ Although ABH mice express normal complement components, unlike some laboratory mice, and can promote complement‐mediated lysis by antibodies,^35^, ^36^ ABH mice have abnormal Fc receptors that could influence function.^37^ This may account for subtle differences in depletion kinetics between mouse strains.^19^ However, if humans do not show marked depletion of B cells in their lymphoid tissue, and possibly bone marrow, this could facilitate the rapid (hyper)repopulation of the peripheral blood after alemtuzumab treatment.^11^ This may contribute to the development of secondary B‐cell autoimmune diseases that are common following treatment of people with MS, who are genetically prone to autoimmunity, with alemtuzumab.^11^, ^12^, ^13^ This does not occur with similar frequency in people with cancer, when they are treated with alemtuzumab.^38^

We have shown also that a transient depletion of CD4 T cells followed by tolerogenic delivery^15^ of pathogenic antigen can induce robust unresponsiveness that effectively silences established, relapsing disease.^4^ This acts via multiple mechanisms that involve central and peripheral deletion, anergy and also active suppression of T‐ and B‐cell responses, as shown previously.^4^, ^15^, ^21^ CD52 lymphocyte depletion was used to determine whether CD52‐specific mAb had value as a depleting agent, either as a prelude to antigen‐specific therapy to inhibit MS, or as a method to induce antigen‐specific tolerance to limit secondary autoimmune diseases. However, it appeared that rather than facilitating the induction of unresponsiveness, CD52 depletion was actually inhibiting tolerogenic mechanisms. This appeared to occur through inhibition of a CD8 T‐cell function as additional depletion of CD8^+^, but not CD20^+^, cells largely inhibited the tolerogenic potential of CD4‐specific depleting antibodies. Although there is much interest in the pathogenic potential of CD8^+^ T cells in MS, it is well established that this subset has regulatory activity that may control autoimmunity.^39^, ^40^

Furthermore, although it has been reported that alemtuzumab induces a relative sparing of Fox3P positive, T regulatory cells as shown here, which favours the regulation of CD4 T cells,^41^, ^42^ in MS there is a substantial reduction in the absolute number of regulatory T cells and CD8^+^ T cells.^11^, ^41^ Hence, following alemtuzumab treatment, B cells repopulate in the relative absence of CD4 T regulatory cells and CD8 T suppressor cells. These T regulatory cells can control silencing of autoreactive immature B cells exiting the bone marrow^43^, ^44^ and furthermore T cells control the affinity maturation of antibodies within lymphoid tissue that may lead to B‐cell autoreactivity.^45^, ^46^ The loss of immune‐tolerance and B‐cell hyper‐repopulation,^11^ may underlie the high prevalence (about 50% within a median of 7 years^13^) of B‐cell autoimmune diseases occurring as a consequence of alemtuzumab treatment.^13^, ^14^ Furthermore, it may contribute also to the marked occurrence of alemtuzumab‐specific antibodies that occur in > 80%, higher than seen with other humanized antibodies,^47^ of people treated with alemtuzumab.^22^, ^23^ Although these do not appear to eliminate immune depletion,^23^ the development of binding or neutralizing antibodies could impact the therapeutic activity of the antibody in people requiring repeated infusions that are needed in some people with MS.^13^ In this study, B‐cell hyper‐proliferation and anti‐globulin responses to the mCD52 mAb were not detected; however, although ABH mice are susceptible to a variety of different induced autoimmune diseases and can produce high titre autoantibodies,^48^ performing studies in animals that are genetically prone to spontaneous B‐cell autoimmune diseases, may be more suitable to try and model mCD52 mAb‐induced secondary autoimmunity. Likewise, further studies are warranted to define the CD8 T‐cell regulatory mechanisms.

Interestingly, B‐cell hyper‐repopulation and marked loss of T cells does not occur following B‐cell depletion with hCD20‐specific antibodies or cladribine in people with MS, both of which control relapsing MS, but are not associated with the development of secondary autoimmune diseases.^49^, ^50^ They do however occur in non‐alemtuzumab‐induced, non‐ablative haematopoietic stem cell therapy, which is also associated with delayed onset of B‐cell autoimmune diseases.^51^ Therefore, B‐cell repopulation and perhaps autosensitization occur in an environment that is deficient in T‐cell regulation and allows autoimmunity to be initiated. This can then become manifest once CD4^+^ T‐cell help is repopulated to levels that can facilitate autoantibody production and so account for a relative delay in the development of autoimmunity relative to the B‐cell repopulation kinetics.^11^, ^12^, ^13^ As people taking alemtuzumab require regular monthly blood sampling as part of their care package, monitoring the development of antigen‐specific (such as thyroid or, more easily, alemtuzumab‐specific) B‐cell and T‐cell function may provide a useful model system to study the development of human autoimmunity. Furthermore, methods to limit B‐cell hyper‐population or promote reconstitution of CD8 T‐cell regulation may provide a method to limit the unwanted adverse effects of this very effective treatment of multiple sclerosis.

## Grant support

The authors would like to thank the support of the Medical Research Council, the Multiple Sclerosis Society and work relating to CD52 was supported by Genzyme Corporation, a Sanofi company.

## Disclosures

Work was supported in part by Sanofi Genzyme to DB. GG has undertaken paid consultancy and provided lectures on behalf of Genzyme, a Sanofi company. Although considered to be irrelevant, while SVK has nothing to declare, DB is a founder and consultant to Canbex therapeutics and has also received research funds from Canbex therapeutics. GP is a shareholder of Canbex therapeutics. GG has received fees for participation in advisory boards for AbbVie Biotherapeutics, Biogen, Canbex, Ironwood, Novartis, Merck, Merck Serono, Roche, Sanofi Genzyme, Synthon, Teva and Vertex; speaker fees from AbbVie, Biogen, Bayer HealthCare, Genzyme, Merck Serono, Sanofi‐Aventis and Teva; and research support from Biogen, Genzyme, Ironwood, Merck, Merck Serono and Novartis.

## Supporting information


**Figure S1.** CD4‐binding epitopes.
**Table S1.** CD4‐binding epitopes.
