# Supplementary material for: Depletion of CD52‐positive cells inhibits the development of central nervous system autoimmune disease, but deletes an immune‐tolerance promoting CD8 T‐cell population. Implications for secondary autoimmunity of alemtuzumab in multiple sclerosis
Source: Immunology. 2017 Jan 3;150(4):444–55. doi: 10.1111/imm.12696 (PMC5343359; doi:10.1111/imm.12696)
Supplement: Supplementary file 1 — Figure S1. CD4‐binding epitopes. Table S1. CD4‐binding epitopes. [file IMM-150-444-s001.docx]

**Supplementary Data**

**Supplementary Figure 1S.** *CD4-binding epitopes*


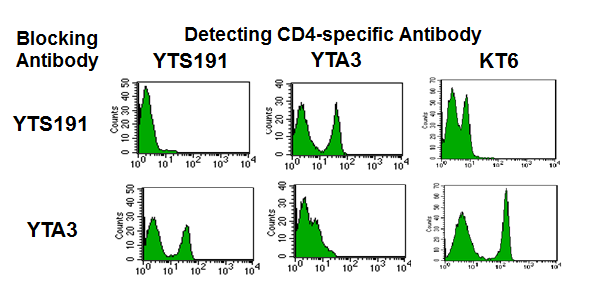


1 x 10^6^ splenocytes were incubated with 2µg unlabelled CD4-specific (YTS191.1.1 or YTA3.1.1) antibody for 30-60minutes prior to incubation with 0.5 µg fluorescent YTS191.1, YTA3.1 or KT6 antibodies for 30minutes and the binding was analysed by flow cytometry. Binding was present or was blocked (completely or partially with reduced fluorescence intensity).

**Supplementary Table 1S.** *CD4-binding epitopes*

|  | Clone | Binding of Detecting Fluorescent CD4-specific mAb | | | | | |
| --- | --- | --- | --- | --- | --- | --- | --- |
|  |  | YTS191.1 | GK1.5 | RM4-5 | RM4-4 | YTA3.1 | KT-174 |
| Coating  CD4-T cell  Specific  mAb | YTS191.1 | Blocked | Blocked | Blocked | Binding | Binding | Binding |
|  | GK1.5 | Blocked | Blocked | Not Tested | Not Tested | Not Tested | Not Tested |
|  | RM4-5 | Blocked | Blocked | Blocked | Binding | Not Tested | Not Tested |
|  | RM4-4 | Binding | Binding | Binding | Blocked | Binding | Not Tested |
|  | YTA3.1 | Binding | Binding | Binding | Binding | Blocked | Binding |

1 x 10^6^ splenocytes were incubated with 2µg unlabelled CD4-specific antibody for 30-60minutes prior to incubation with 0.5 µg fluorescent antibodies for 30minutes and the binding was analysed by flow cytometry. Binding was present or was blocked (completely or partially with reduced fluorescence intensity) (n=3).
